# Ethylene, a key factor in the regulation of seed dormancy

**DOI:** 10.3389/fpls.2014.00539

**Published:** 2014-10-10

**Authors:** Françoise Corbineau, Qiong Xia, Christophe Bailly, Hayat El-Maarouf-Bouteau

**Affiliations:** Biologie des Semences (Seed Biology), UMR7622 CNRS-UPMC, Sorbonne Universités – Université Pierre et Marie Curie-ParisParis, France

**Keywords:** abscisic acid, dormancy, ethylene, gibberellins, reactive oxygen species, seed germination

## Abstract

Ethylene is an important component of the gaseous environment, and regulates numerous plant developmental processes including seed germination and seedling establishment. Dormancy, the inability to germinate in apparently favorable conditions, has been demonstrated to be regulated by the hormonal balance between abscisic acid (ABA) and gibberellins (GAs). Ethylene plays a key role in dormancy release in numerous species, the effective concentrations allowing the germination of dormant seeds ranging between 0.1 and 200 μL L^-1^. Studies using inhibitors of ethylene biosynthesis or of ethylene action and analysis of mutant lines altered in genes involved in the ethylene signaling pathway (*etr1*, *ein2*, *ain1*, *etr1*, and *erf1*) demonstrate the involvement of ethylene in the regulation of germination and dormancy. Ethylene counteracts ABA effects through a regulation of ABA metabolism and signaling pathways. Moreover, ethylene insensitive mutants in *Arabidopsis* are more sensitive to ABA and the seeds are more dormant. Numerous data also show an interaction between ABA, GAs and ethylene metabolism and signaling pathways. It has been increasingly demonstrated that reactive oxygen species (ROS) may play a significant role in the regulation of seed germination interacting with hormonal signaling pathways. In the present review the responsiveness of seeds to ethylene will be described, and the key role of ethylene in the regulation of seed dormancy via a crosstalk between hormones and other signals will be discussed.

## INTRODUCTION

In soil, seeds are exposed to various environmental factors including temperature, moisture, oxygen and light, which regulate seed germination, and subsequent seedling growth. Phase I of the germination process is initiated by imbibition, which is required to activate the respiratory metabolism, and transcriptional and translational activities. In phase II called germination *sensu stricto*, water uptake ceases and reserve mobilization starts. Phase III is characterized by radicle protrusion (Bewley and Black, 1994; Bewley, 1997; Nonogaki et al., 2010; Weitbrecht et al., 2011). Germination requires specific temperatures, oxygen levels and light, the exact proportions being species specific. However, the seeds of species (or even within species) do not germinate, or do so with difficulty, even when incubated under apparently favorable conditions; these are considered as dormant and cannot germinate in the same conditions (i.e., water, air, temperature) under which non-dormant seeds do (Bewley and Black, 1994; Corbineau and Côme, 1995; Bewley, 1997). Dormancy is a heritable trait, but its intensity at harvest and its maintenance after harvest is highly modulated by the environmental conditions throughout seed development and ripening on the plant, and during seed storage (Bewley, 1997). Factors inhibiting germination can reside within the embryo itself (embryo dormancy) or can result from an inhibitory action of the covering structures (seed coat imposed dormancy; Bewley and Black, 1994; Hilhorst, 2007). Primary dormancy sets during seed development, but a secondary dormancy can develop in mature seeds with some degree of primary dormancy or in non-dormant seeds in response to unfavorable conditions for germination (Hilhorst, 2007; Hilhorst et al., 2010).

The involvement of the hormonal balance between abscisic acid (ABA) and gibberellins (GAs) in the regulation of seed germination and dormancy in response to environmental signals is well documented and discussed in recent reviews (Finkelstein et al., 2008; Nambara et al., 2010; Nonogaki et al., 2010; Weitbrecht et al., 2011; Graeber et al., 2012; Rajjou et al., 2012; Arc et al., 2013; Miransari and Smith, 2014). ABA is well known to play a crucial role in induction of dormancy in the developing seeds and in maintenance of dormancy during seed imbibition, while GAs are involved in dormancy release and/or germination (Finkelstein et al., 2008; Cutler et al., 2010; Nambara et al., 2010; Miransari and Smith, 2014). In addition to GAs and ABA, other hormones (ethylene, jasmonates, auxins) also play a role in the control of seed germination (Linkies and Leubner-Metzger, 2012; Arc et al., 2013; Miransari and Smith, 2014). Ethylene (C_2_H_4_) in particular regulates germination and dormancy of numerous species via a complex hormonal signaling network (Matilla, 2000; Brady and McCourt, 2003; Feurtado and Kermode, 2007; Matilla and Matilla-Vazquez, 2008; Arc et al., 2013).

The role of reactive oxygen species (ROS) in seed biology has progressively emerged and evolved this last decade. Originally considered as harmful compounds, causing deleterious reactions toward a wide range of biomolecules and thus to seeds, ROS are now widely acknowledged as signaling compounds regulating the germination process through an hormonal network (Bailly et al., 2008; Diaz-Vivancos et al., 2013; El-Maarouf-Bouteau et al., 2014).

In this review, we describe how ethylene interacts with other plant hormones in regulation of germination and dormancy, concentrating on its interactions with ABA, GAs, and ROS.

## ETHYLENE BIOSYNTHESIS DURING GERMINATION

Ethylene production by seeds begins immediately after the onset of imbibition and increases with time of germination. There is, however, a peak in ethylene emission concomitant with the radicle protrusion through the seed coat (Ketring and Morgan, 1969; Fu and Yang, 1983; Satoh and Esashi, 1983; Gallardo et al., 1991; Gorecki et al., 1991; Siriwitayawan et al., 2003; El-Maarouf-Bouteau et al., 2014). Seed ethylene production is species dependent (Kepczynski and Kepczynska, 1997; Matilla, 2000), but is generally below levels detectable by gas chromatography during imbibition. Using a high sensitivity laser photo acoustic spectroscopy (Cristescu et al., 2008), El-Maarouf-Bouteau et al. (2014) have confirmed the occurrence of an ethylene peak at the end of the germination process in sunflower (*Helianthus annuus*) seeds. Interestingly, a close relationship between the ability to produce ethylene and seed vigor has been reported in various species including rape (*Brassica napus*; Takayanagi and Harrington, 1971), cotton (*Gossypium spp*.; Ketring et al., 1974), peanut (*Arachis hypogaea*; Ketring et al., 1974), cocklebur (*Xanthium pennsylvanicum*; Gorecki et al., 1991), snap bean (*Phaseolus vulgaris*; Samimy and Taylor, 1983), sunflower (Chonowski et al., 1997) and pea (*Pisum sativum*; Gorecki et al., 1991), and 1-aminocyclopropane 1-carboxylic acid (ACC)-dependent C_2_H_4_ production was proposed as a marker of seed quality (Khan, 1994; Corbineau, 2012).

**FIGURE 1:**
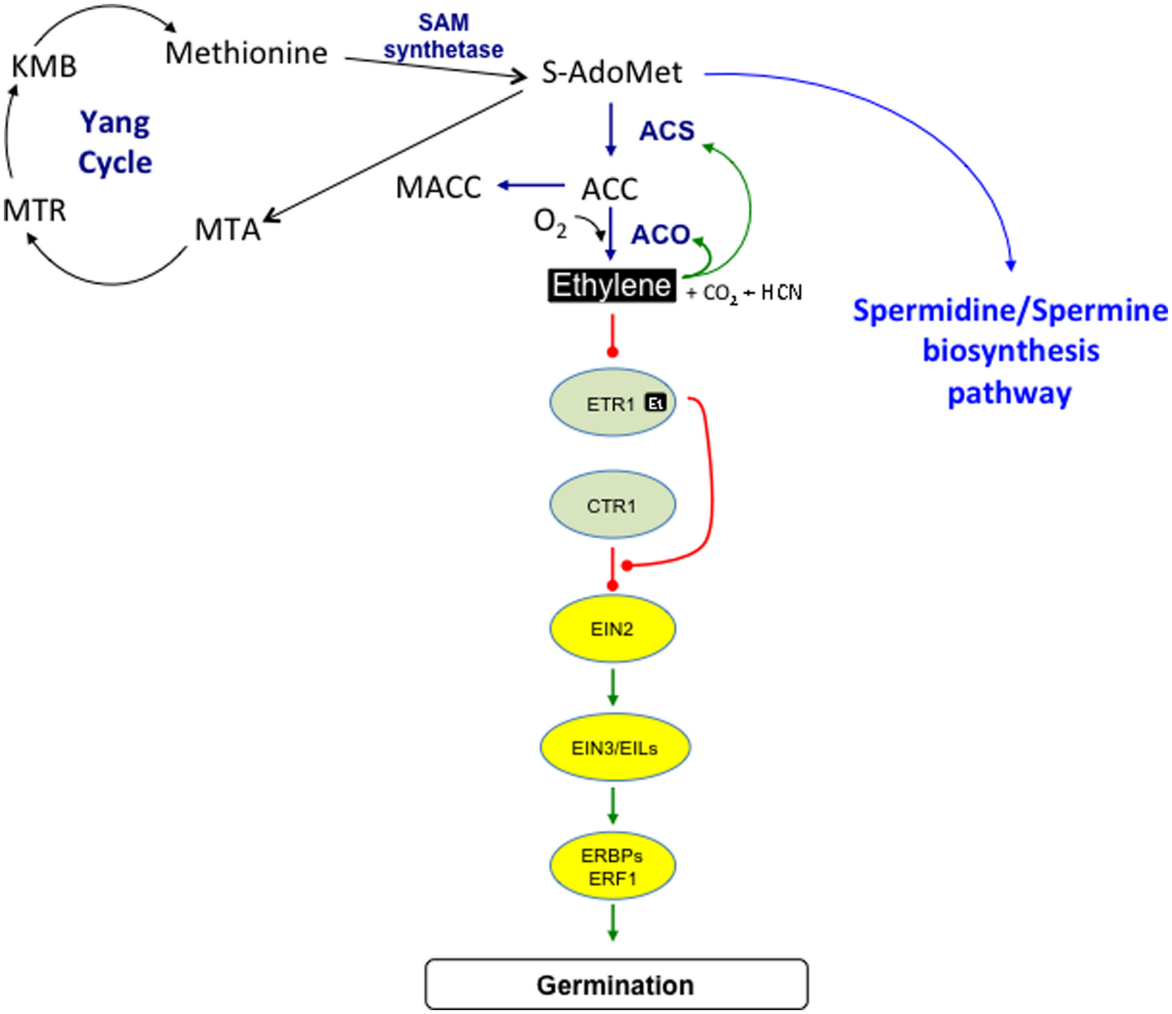
**Ethylene biosynthesis and signaling pathways.** *S*-adenosyl methionine (*S*-AdoMet) is synthesized from methionine by the SAM synthetase, it is then converted to 1-aminocyclopropane-1-carboxylic acid (ACC) by the ACC synthase (ACS), 5-methylthioadenosine (MTA) being a by-product. MTA is recycled to methionine through the Yang Cycle by successive enzymatic reactions involving different intermediates among which 5-methylthioribose (MTR) and 2-keto-4-methylthiobutyrate (KMB). *S*-AdoMet is also the precursor of the spermidine/spermine biosynthesis pathway. Ethylene production results from the ACC oxidation catalyzed by the ACC oxidase (ACO) that also generates carbon dioxide and cyanide. Malonylation of ACC to malonyl-ACC (MACC) reduces ACC content and consequently ethylene production. Ethylene can stimulate its own biosynthesis, by improving ACC synthesis catalyzed by ACS, and conversion to ethylene by ACO. Ethylene binds to receptors (among which ethylene receptor 1, ETR1) located in the endoplasmic reticulum, which leads to the deactivation of the receptors that become able to recruit CTR1 (constitutive triple response). Release of CTR1 inhibition allows EIN2 to act as a positive regulator of ethylene signaling pathway. EIN2 acts upstream of nuclear transcription factors, such as EIN3 (ethylene insensitive), EILs (EIN3-like), ERBPs (ethylene responsive element binding protein), and ERFs (ethylene response factor). → and —∙ arrows indicate positive and negative interactions between the different elements of the signaling cascade, respectively.

The pathway of ethylene biosynthesis in seeds is the same as that described for other plant organs, in which *S*-adenosyl-methionine (*S*-AdoMet) and ACC are the main intermediates (Yang and Hoffman, 1984; Wang et al., 2002; Rzewuski and Sauter, 2008; **Figure 1**). *S*-AdoMet synthesized from methionine by the *S*-AdoMet synthetase (or SAM synthetase), is converted to ACC, the direct precursor of ethylene, by ACC synthase (*S*-adenosyl-L-methionine methylthioadenosine-lyase, ACS). The by-product 5′-methylthioadenosine (MTA) is recycled back to methionine through the Yang Cycle (Yang and Hoffman, 1984; Kende, 1993). *S*-AdoMet is also the precursor of the biosynthesis of polyamines, which can also play a role in seed germination (Matilla, 1996). Ethylene production results from the oxidation of ACC by ACC oxidase (ACO), which also generates CO_2_ and hydrogen cyanide (HCN; **Figure 1**). Autocatalytic synthesis of ethylene *via* induction of *ACC* and *ACO* transcription is well known in fruit ripening (Lin et al., 2009), ethylene also regulates *ACO* expression in pea (Petruzzelli et al., 2000, 2003), beechnut (*Fagus sylvatica*; Calvo et al., 2004b), and turnip (*Brassica rapa*; Puga-Hermida et al., 2003). In contrast, ethylene or ACC does not affect the abundance of *ACO* transcript in sugar beet (*Beta vulgaris*; Hermann et al., 2007) and, expression of *SoACS7* in *Sisymbrium officinale* and *PsAC1* in pea (Petruzzelli et al., 2000, 2003; Iglesias-Fernandez and Matilla, 2010).

Increased ethylene production during germination is associated with an increase in ACO activity, as well as a progressive accumulation of *ACS* and *ACO* transcripts (Gomez-Jimenez et al., 1998; Matilla and Matilla-Vazquez, 2008; Linkies et al., 2009; Iglesias-Fernandez and Matilla, 2010; Linkies and Leubner-Metzger, 2012). Although ACS is considered to be a key enzyme in the regulation of ethylene production in most plant responses to abiotic and biotic stresses (Wang et al., 2002), it was demonstrated in seeds that ACO activity plays a fundamental role during germination (Matilla and Matilla-Vazquez, 2008; Linkies and Leubner-Metzger, 2012). Both ACS and ACO are encoded by a multigene family, and the regulation of particular *ACS* and *ACO* genes differ among each other (Wang et al., 2002, 2005; Yamagami et al., 2003). In both *Arabidopsis* and cress (*Lepidium sativum*), *ACO1* and *ACO2* have been demonstrated to be the major *ACOs* involved in ethylene synthesis (Linkies et al., 2009; Linkies and Leubner-Metzger, 2012), and the correlation between the abundance of *ACO* transcripts and the ACO activity suggests its regulation at a transcriptional level during germination.

Ethylene is involved in various developmental processes and responses to biotic and abiotic stresses in plants (Bleecker and Kende, 2000; Wang et al., 2002). The key components in its signaling pathway have been identified using a molecular dissection of ethylene responsiveness in *Arabidopsis* (Wang et al., 2002; Stepanova and Alonso, 2009; Yoo et al., 2009). Five membrane-localized ethylene receptors, ethylene resistant 1 (ETR1), ETR2, ethylene response sensor 1 (ERS1), ERS2, and ethylene insensitive 4 (EIN4) exist in *Arabidopsis* (Wang et al., 2002). Among them, ETR1 and ERS1 contain three transmembrane domains in the N-terminus and a histidine kinase domain in the C-terminus, when ETR2, EIN4, and ERS2 present four transmembrane regions and a serine-threonine kinase domain in the C-terminus (Wang et al., 2006; Kendrick and Chang, 2008). Binding of ethylene to its receptors results in inactivation of CTR1 (constitutive triple response 1) protein kinase, which in turn activates the kinase cascade controlling EIN2 and its transcription factors in the nucleus. These, such as EIN3, EILs, ethylene response element binding proteins (EREBPs)/ethylene responsive factors (ERFs) activate the transcription of ethylene response genes (Wang et al., 2002; Liu et al., 2004; Hall et al., 2007; Rzewuski and Sauter, 2008; Yoo et al., 2008, 2009; Stepanova and Alonso, 2009; **Figure 1**). Recently, it was demonstrated that EIN2 is phosphorylated by CTR1 kinase in the absence of ethylene, and that EIN2 protein level is regulated through its degradation by the proteasome (Qiao et al., 2009, 2012; Ju et al., 2012).

## SEED RESPONSIVENESS TO EXOGENOUS ETHYLENE

Exogenous ethylene or ethephon, an ethylene releasing compound, improves germination in numerous species (Esashi, 1991; Corbineau and Côme, 1995; Kepczynski and Kepczynska, 1997; Matilla, 2000; Matilla and Matilla-Vazquez, 2008; Arc et al., 2013). It stimulates germination of non-dormant seeds incubated in non-optimal environmental conditions such as too high temperatures (Rao et al., 1975; Abeles, 1986; Gallardo et al., 1991), osmotic stress (Negm and Smith, 1978; Kepczynski, 1986; Khan et al., 2009), hypoxia (Esashi et al., 1989; Corbineau and Côme, 1992), and salinity (Zapata et al., 2003; Wang et al., 2011; Lin et al., 2013; Silva et al., 2014). It can also break primary and secondary dormancy (**Table 1**). It breaks the embryo dormancy in apple (*Malus domestica*; Kepczynski et al., 1977; Sinska and Gladon, 1984; Sinska and Lewandowska, 1991) and beechnut (Calvo et al., 2004a), the dormancy of which is usually broken by chilling, and in sunflower (Corbineau et al., 1990), the dormancy of which is progressively alleviated during dry storage (after-ripening). It also promotes the germination of seeds exhibiting a seed coat imposed dormancy in various species such as cocklebur (Katoh and Esashi, 1975; Esashi et al., 1978), subterranean clover (*Trifolium subterraneum*; Esashi and Leopold, 1969), *Rumex crispus* (Taylorson, 1979), and *Arabidopsis* (Siriwitayawan et al., 2003). In particular, it can also overcome the secondary dormancy induced by high temperatures in lettuce (*Lactuca sativa*; Speer et al., 1974; Abeles, 1986), sunflower (Corbineau et al., 1988), *Amaranthus caudatus* (Kepczynski et al., 1996a) and *Amaranthus paniculatus* (Kepczynski and Kepczynska, 1993). In *Rhus coriaria*, a post-fire pioneer, low ethylene concentrations (0.03–0.10 μL L^-1^) released by wet ash stimulates germination (Ne’eman et al., 1999), although it does not improve that of many other species in which germination is smoke-induced (Brown and van Staden, 1997). Ethylene also improves germination of seeds from parasitic plants such as *Striga asiatica, Striga lutea* and *Striga hermonthica* (Egley and Dale, 1970; Bebawi and Eplee, 1986).

**Table 1:** Plant species whose seed dormancy is broken by ethylene, ethephon, an ethylene releasing compound, or 1-aminocyclopropane-1-carboxylic acid, the direct precursor of ethylene.

Type of dormancy	Species	Reference
Primary dormancy	*Amaranthus caudatus* *Amaranthus retroflexus* *Arabidopsis thaliana* *Arachis hypogaea* *Chenopodium album* *Fagus sylvatica* *Helianthus annuus* *Pyrus malus* *Rhus coriaria* *Rumex crispus* *Stylosanthes humilis* *Trifolium subterraneum* *Xanthium pennsylvanicum*	Kepczynski and Karssen (1985) Kepczynski et al. (1996b) Siriwitayawan et al. (2003) Ketring and Morgan (1969) Machabée and Saini (1991) Calvo et al. (2004a) Corbineau et al. (1990) Kepczynski et al. (1977) Sinska and Gladon (1984) Ne’eman et al. (1999) Taylorson (1979) Ribeiro and Barros (2006) Esashi and Leopold (1969) Katoh and Esashi (1975)

Thermo-dormancy	*Lactuca sativa*	Speer et al. (1974)

Secondary dormancy	*Amaranthus caudatus* *Amaranthus paniculatus* *Helianthus annuus* *Lactuca sativa* *Rumex crispus* *Xanthium pennsylvanicum*	Kepczynski and Karssen (1985) Kepczynski and Kepczynska (1993) Corbineau et al. (1988) Abeles (1986) Samimy and Khan (1983) Esashi et al. (1978)

The stimulatory effect of ethylene is dose dependent, the hormone being efficient when applied at concentration ranging from 0.1 to 200 μL L^-1^ depending on the species, the depth of dormancy and the environmental conditions. Breaking of dormancy either during chilling in apple (Sinska, 1989) or dry storage in sunflower (Corbineau and Côme, 2003), *Amaranthus retroflexus* (Kepczynski and Sznigir, 2014) and *Stylosanthes humilis* (Ribeiro and Barros, 2006) is associated with an increasing sensitivity to ethylene. At harvest, dormant sunflower seeds require 50 μL L^-1^ ethylene in order to germinate at 15°C, but only 10 and 3 μL L^-1^ after 8 and 15 weeks of dry-storage at 5°C, respectively (Corbineau and Côme, 2003). In contrast, the responsiveness to the hormone decreases progressively during seed incubation under environmental conditions that induce a secondary dormancy (Negm et al., 1973; Speer et al., 1974; Esashi et al., 1978; Jones and Hall, 1984; Corbineau and Côme, 2003).

Although ethylene stimulates the germination of numerous light sensitive seeds, it does not overcome the light requirement in *Amaranthus retroflexus* (Schönbeck and Egley, 1981), celery (*Apium graveolens*; Thomas et al., 1975), lettuce (Burdett and Vidaver, 1971), and *Spergula arvensis* (Olatoye and Hall, 1973). Recently, Wilson et al. (2014b) demonstrate that loss of ETR1 reduces the inhibitory effect of far-red on the germination of *Arabidopsis* seeds through expression of genes involved in ABA and GAs metabolism. An epistasis analysis performed by the same authors also suggests that ETR1 may interact with phytochromes to control seed germination.

An additive or synergistic effect of CO_2_ and ethylene has also been demonstrated in seeds of peanut (Ketring and Morgan, 1972), *Spergula arvensis* (Jones and Hall, 1984), cocklebur (Katoh and Esashi, 1975), and lettuce (Negm and Smith, 1978; Saini et al., 1986). In the case of cocklebur (Esashi et al., 1986) and sunflower (Corbineau et al., 1990) seeds, it was suggested that the improving effect of CO_2_ results from an enhancement of C_2_H_4_ biosynthesis, since it is suppressed in the presence of inhibitors of ethylene synthesis.

## INVOLVEMENT OF ETHYLENE IN SEED GERMINATION AND DORMANCY

Numerous studies demonstrate that the ability to germinate correlates with ethylene production, suggesting that ethylene is involved in the regulation of seed germination and dormancy (reviewed in Kepczynski and Kepczynska, 1997; Matilla and Matilla-Vazquez, 2008; Arc et al., 2013). For example, induction of thermodormancy at high temperatures is associated with a reduced ethylene production in chickpea (*Cicer arietinum*; Gallardo et al., 1991), sunflower (Corbineau et al., 1988), and lettuce (Prusinski and Khan, 1990). This decrease in C_2_H_4_ production may result from an increase in ACC-malonyltransferase activity, thus from a decrease in ACC content as demonstrated in chickpea (Martinez-Reina et al., 1996), an inhibition of ACO activity (Corbineau et al., 1988; Gallardo et al., 1991), or a reduced expression of *ACS* and *ACO* (Argyris et al., 2008). In contrast, breaking of dormancy by various treatments (e.g., chilling, GAs, NO, HCN) leads to an increase in ethylene production (Kepczynski and Kepczynska, 1997; Arc et al., 2013). In *Arabidopsis*, the inductive effect of chilling is associated with a reduced expression of *ACO*, but in a transient induction of *ACS* (Narsai et al., 2011; Linkies and Leubner-Metzger, 2012). However, after-ripening of *Sisymbrium officinale* seeds inhibits expression of *SoACS7* and *SoACO2* which are involved in ethylene biosynthesis, during early seed incubation, but stimulates that of *SoGA20ox2, SoGA3ox2, and SoGA2ox6* involved in GA metabolism (Iglesias-Fernandez and Matilla, 2009).

Data obtained using inhibitors of ethylene biosynthesis pathway or mutants altered in ethylene biosynthesis and signaling pathways demonstrated that endogenous ethylene plays a key role in the regulation of germination and dormancy. Incubation of seeds in the presence of aminoethoxyvinylglycine (AVG) and aminooxyacetic acid (AOA), inhibitors of ACS activity, CoCl_2_ and α-aminoisobutyric acid (α-AIB), inhibitors of ACO activity, or 2,5-norbornadiene (2,5 NBD) and silver thiosulfate (STS), inhibitors of ethylene action, allowed demonstration of the involvement of endogenous ethylene in germination and dormancy breakage (Kepczynski et al., 1977, 2003; Sinska and Gladon, 1989; Corbineau et al., 1990; Longan and Stewart, 1992; Gallardo et al., 1994; Hermann et al., 2007). In contrast, seed incubation in the presence of ACC, the direct precursor of ethylene, improves seed germination in numerous species such as lettuce (Fu and Yang, 1983), sunflower (Corbineau et al., 1990), cocklebur (Satoh et al., 1984), *Amaranthus sp.* (Kepczynski, 1986; Kepczynski et al., 1996b), chickpea (Gallardo et al., 1994), and sugar beet (Hermann et al., 2007). This effect of ACC suggests that ACO is potentially active, and that dormancy might result from insufficient ACC level due to low ACS activity.

It is important to notice that HCN, a co-product of ACC oxidation, can also break seed dormancy in apple (Perino et al., 1984; Lewak, 2011; Krasuska and Gniazdowska, 2012; Krasuska et al., 2014), sunflower (Oracz et al., 2008) and *Amaranthus retroflexus* (Kepczynski and Sznigir, 2014).

Using *Arabidopsis* lines altered in ethylene biosynthesis and signaling allowed to characterize the regulation of dormancy by ethylene (**Table 2**). Seeds of ethylene insensitive *etr1* (*ethylene resistant*) as well as *ein2* (*ethylene insensitive 2*) mutants display enhanced primary dormancy relative to wild type, probably due to high ABA sensitivity, whereas *ctr1* (*constitutive triple responses*) mutant have a slightly enhanced rate of germination (Bleecker et al., 1988; Leubner-Metzger et al., 1998; Beaudoin et al., 2000; Ghassemian et al., 2000; Hall et al., 2001; Chiwocha et al., 2005; Subbiah and Reddy, 2010). EIN2 plays a key role in the ethylene signaling pathway, and loss of its function results in a hypersensitivy to salt and osmotic stress during germination and early seedling development in *Arabidopsis* (Wang et al., 2007). *ERFs* genes might also play a pivotal role in ethylene responsiveness and regulation of germination (Leubner-Metzger et al., 1998; Pirrello et al., 2006). *FsERF1* expression is minimal in dormant beechnut embryo, but increases during moist chilling which breaks dormancy (Jimenez et al., 2005). In sunflower, *ERF1* expression is fivefold higher in non-dormant than in dormant embryos, and expression is markedly stimulated by HCN, which breaks dormancy (Oracz et al., 2008). In tomato (*Solanum lycopersicon*), *SlERF2* transcript abundance is higher in germinating seeds than in non-germinating ones, and its overexpression in transgenic lines results in premature seed germination (Pirrello et al., 2006).

**Table 2:** Dormancy and ABA sensitivity of various mutants of *Arabidopsis thaliana* affected in ethylene biosynthesis or signaling pathway.

Mutant or transgenic lines^a^	Gene/locus	Seed dormancy	Hormone sensitivity and content	Reference^b^
*etr1-1* *etr1-2*	*ETR1*	Enhanced	C_2_H_4_ insensitive and ABA hypersensitive Higher ABA content	1, 2, 3, 4, 6, 7, 8, 9, 10, 11, 12
*etr1-3*	*ETR1*	Enhanced	Reduced C_2_H_4_ sensitivity	10
*etr1-6*	*ETR1*	Slightly enhanced	More sensitive to ABA	10, 13
*etr1-8*	*ETR1*	Enhanced	–	10

*ein2-1, ein2-5, ein2-49*	*EIN2*	Enhanced	ABA hypersensitivity Higher ABA content	1, 3, 4, 7, 11, 12
*ein4-4*	*EIN4*	Enhanced	–	10
*ein6*	*EIN6*	Enhanced	ABA hypersensitivity	11

*ctr1-1, ctr1-10*	*CTR1*	Early germination	Reduced ABA sensitivity	1, 3, 7, 9, 11, 12

*acs7*	*ACS*	Early germination	ABA hypersensitivity	5

*eto3*		Early germination	Reduced ABA sensitivity	3, 11

*^a^mutant abbreviations: acs7 = ACC synthetase7; ctr1 = constitutive triple response1; ein2, ein4, ein6 = ethylene insensitive1, 4, 6; etr1 = ethylene resistant1; eto3 = ethylene overproducer3.*

*^b^references: (1) Beaudoin et al. (2000); (2) Bleecker et al. (1988); (3) Cheng et al. (2009); (4) Chiwocha et al. (2005); (5) Dong et al. (2011); (6) Ghassemian et al. (2000); (7) Hall et al. (2001); (8) Leubner-Metzger et al. (1998); (9) Ouaked et al. (2003); (10) Siriwitayawan et al. (2003); (11) Subbiah and Reddy (2010); (12) Wang et al. (2007); (13) Wilson et al. (2014a).*

Transcriptional arrays have been used to draw a global view of gene expression in germinating and non-germinating seeds and core sets of genes were analyzed with respect to hormone responsive elements. The analysis of transcriptome data of dormant and after-ripened states in *Arabidopsis* performed by Cadman et al. (2006) showed that *ACS2* gene expression was up-regulated in the dormant state and *AtERF5* was up-regulated in germinating state. In lettuce, Argyris et al. (2008) have shown that ethylene responsive genes are regulated by thermo-inhibition; *ACO* and *ACS* gene expression is reduced while *CTR1, EIN2, ETR1* expression is increased at high temperature. These results point out the gap that exists between hormone metabolism and signaling regulation at the level of gene expression. In wheat seeds, 78 probesets annotated as ethylene metabolism and signaling genes have been differentially expressed between dormant and after-ripened seeds (Chitnis et al., 2014). *ACO* is represented by four probesets that are up-regulated in after-ripened wheat seeds but no *ACS* corresponding probeset has been found. Ethylene signaling element as reversion to ethylene sensitivity 1, *ERS1, EBF1* (EIN3 binding box protein1), prohibitin 3 or *ERF* have been shown to be up-regulated in after-ripened seeds at 12 or 24 h of imbibition. The number of genes related to ethylene involved in germination is underestimated since only direct known ethylene signaling components are targeted for analysis in omic studies. It has been shown that treatment with ACC of 7 days germinated seedlings triggers change in expression of 544 genes, among them 244 were common to seeds given an ABA treatment (Nemhauser et al., 2006). These results have been used to compare genes regulated in *Lepidium* seed tissues during germination (Linkies et al., 2009). *Pectate Lyase 1*, *Argos-like*, *Expansin A2*, *B-1,3-glucanase* and *chitinase B* which play an important role in endosperm weakening and/or radicle growth in germination of *Lepidium* seeds, are proposed as putative ethylene response down-stream genes. Cell wall loosening enzymes expressed in endosperm are also controlled by both ABA and GA (Groot et al., 1988; Toorop et al., 2000).

## ETHYLENE CROSSTALK WITH ABA/GAs AND SEED GERMINATION

### INTERRELATIONSHIP BETWEEN ETHYLENE AND ABA

The antagonistic effects of ABA and ethylene in the regulation of seed germination and dormancy have been extensively studied (Leubner-Metzger et al., 1998; Beaudoin et al., 2000; Kucera et al., 2005; Matilla and Matilla-Vazquez, 2008; Linkies et al., 2009; Arc et al., 2013). Ethylene overcomes the inhibitory action of ABA on germination of numerous species among which are *Amaranthus caudatus* (Kepczynski, 1986), *Chenopodium album* (Karssen, 1976), cotton (Halloin, 1976), tobacco (*Nicotiana tabacum*), and *Arabidopsis* (Leubner-Metzger et al., 1998). In *Arabidopsis* and *Lepidium sativum*, ethylene also counteracts the inhibition by ABA of endosperm cap weakening and rupture (Linkies et al., 2009). On the contrary, ABA increases the ethylene requirement in order to release dormancy in sunflower (Corbineau and Côme, 1995, 2003) and *Amaranthus caudatus* (Kepczynski et al., 2003). The negative interaction between ABA and ethylene is also supported by data obtained with various mutants affected in the signaling pathway of both hormones. Ethylene insensitive mutants (*etr1, ein2,* and *ein6*) are hypersensitive to ABA, whereas seeds of *ein3*, *ein4, ein5*, and *ein7* germinate normally. Conversely, *eto1*, *eto3,* and *ctr1* mutants (characterized by an increase in C_2_H_4_ production) exhibit a reduced sensitivity to ABA (**Table 2**; Beaudoin et al., 2000; Ghassemian et al., 2000; Subbiah and Reddy, 2010). Loss of function of CRT1 enhances the tolerance to ABA of *abi1-1* seeds (Beaudoin et al., 2000). Genetic approaches, using double mutants obtained by crossing ethylene insensitive mutants (*ctr1*, *ein1, ein3,* and *ein6*) with *aba2* mutant, demonstrate that ABA and C_2_H_4_ may also act in parallel, since they exibit phenotypes resulting from both ABA deficiency and altered ethylene sensitivity (Cheng et al., 2009).

Although ACC and exogenous ethylene do not affect ABA content in *Lepidium sativum* (Linkies et al., 2009) and sugar beet (Hermann et al., 2007), *Arabidopsis* seeds from ethylene insensitive mutants, *etr1* and *ein2*, have a higher ABA content than that of wild type seeds (Kende et al., 1998; Beaudoin et al., 2000; Ghassemian et al., 2000; Chiwocha et al., 2005; Wang et al., 2007). For example, mutation in *ETR1* results in an 8-fold higher ABA content in mature seeds than in wild type, probably due to a decrease in ABA conjugation (Chiwocha et al., 2005). Loss of function of ACS7, one of type 3 ACS with a very short C-terminus and no phosphorylation site, results in reduced C_2_H_4_ emission and hyper-sensitivity to ABA, consequently conferring abiotic stress tolerance to *Arabidopsis* seeds (Dong et al., 2011). ABA accumulation is also associated with stimulation of ABA biosynthesis through an up-regulation of *NCED* and a down-regulation of *CYP707A2* in seeds of the *etr1* mutant (Cheng et al., 2009), or an up-regulation of *NCED3* associated with an up-regulation of *ABA1* in *ein2* seeds (Wang et al., 2007).

Inhibition of germination by ABA is associated with an inhibition of *in vivo* ACO activity and is correlated with a reduction in *ACO* transcript accumulation (Bailly et al., 1992; Petruzzelli et al., 2000, 2003; Linkies et al., 2009), leading to a reduction of ethylene production (Kepczynski and Kepczynska, 1997; Matilla, 2000). In *Arabidopsis*, accumulation of *ACO1* and *ACO2* transcripts during germination is inhibited by ABA, and the high level of *ACO1* transcript in ABA-insensitive mutants suggest a tight regulation of ACO expression by ABA (Penfield et al., 2006; Carrera et al., 2008; Linkies et al., 2009). In *Lepidium sativum*, ABA inhibits expression of both *ACO1* and *ACO2* in the endosperm cap (Linkies et al., 2009). Up-regulation of *ACO* transcript has also been detected by microarray analysis in the *aba2* mutant in *Arabidopsis* (Cheng et al., 2009). In contrast, there is an ABA-mediated up-regulation of ACC accumulation and *ACO* expression in sugar beet seeds (Hermann et al., 2007).

### INTERRELATIONSHIP BETWEEN ETHYLENE AND GAs

Gibberellins improve the germination of dormant seeds in numerous species whose dormancy is broken by ethylene, ethephon, or ACC (c.f. **Table 1**). Both hormones promote the germination of primary dormant seeds of *Arabidopsis* (Ogawa et al., 2003; Siriwitayawan et al., 2003), *Amaranthus retroflexus* (Kepczynski et al., 1996b; Kepczynski and Sznigir, 2014), beechnut (Calvo et al., 2004a,b), apple (Kepczynski et al., 1977; Sinska and Gladon, 1984; Sinska, 1989; Lewak, 2011), and *Sisymbrium officinale* (Iglesias-Fernandez and Matilla, 2010). They also break secondary dormancy in *Rumex crispus* (Samimy and Khan, 1983) and cocklebur seeds (Esashi et al., 1975), and thermodormancy in lettuce achenes (Keys et al., 1975). In *Arabidopsis*, C_2_H_4_ restores the germination of the GA-deficient mutant *ga1-3* (Karssen et al., 1989), and GA_3_ stimulates that of the *etr1* mutant (Bleecker et al., 1988), while no stimulatory effect is noted on the germination of the GA-deficient *gib-1* mutant in tomato (Groot and Karssen, 1987). All these data suggest that GAs and ethylene pathways interact (Brady and McCourt, 2003; Feurtado and Kermode, 2007; Matilla and Matilla-Vazquez, 2008; Miransari and Smith, 2014).

In beechnut, incubation of embryos in the presence of GA_3_ results in an accumulation of ACC and an increase in ACC oxidase activity and C_2_H_4_ production, concomitant with an increased expression of *FsACO1* (Calvo et al., 2004a). Similarly, the improving effect of GA_4_ on the germination of *Arabidopsis ga1-3* mutant seeds is associated with an increase in *AtACO* (Ogawa et al., 2003). Decrease of the expression of *FsACO1* in the presence of paclobutrazol, a GAs biosynthesis inhibitor, confirms that GAs activates the ethylene biosynthesis pathway (Calvo et al., 2004a,b). However, in *Sisymbrium officinale,* Iglesias-Fernandez and Matilla (2010) demonstrate that expression of *SoACS7* and *SoACO2* during germination is inhibited by paclobutrazol, but is not affected by application of either ethrel or GA_4+7_. In addition, the up-regulation of *AtERS1* (*ETHYLENE RESPONSE SENSOR* encoding a member of ethylene receptor family) in *Arabidopsis* in the presence of GA_4_ (Ogawa et al., 2003) and of an *EIN-3 like* in beechnut in the presence of GA_3_ (Lorenzo et al., 2000) suggest an effect of GAs on ethylene response.

Numerous data also suggest that ethylene stimulates seed germination by affecting the GAs biosynthesis or signaling pathway. GA_1_, GA_4_, and GA_7_ strongly accumulate in dry mature seeds of the *etr1-2 Arabidopsis* mutant relative to wild type, and both GA_4_ and GA_7_ contents remain higher than in wild type during the two first days of imbibition (Chiwocha et al., 2005). The changes in GA content during germination suggest that lack of ETR1, i.e., of ethylene signaling pathway, results (i) in alteration of GAs biosynthesis pathway, and (ii) in a requirement for higher levels of GAs than wild type, to promote germination (Chiwocha et al., 2005). In beechnut, expression of *FsGA20ox1*, which is involved in the synthesis of active GAs, remains low in stratified seeds (i.e., non-dormant seeds) and seeds treated with GA_3_ or ethephon, but inhibition of ethylene biosynthesis by AOA (2-aminoxyacetic acid) results in an increase in this transcript indicating the involvement of ethylene in the regulation of GA biosynthesis (Calvo et al., 2004b). Studies of expression of genes involved in GA synthesis (*SoGA3ox2* and *SoGA20ox2*) and degradation (*SoGA2ox6*) during imbibition of *Sisymbrium officinale* seeds in the presence of GA_4+7_, ethylene, and inhibitors of GA synthesis or ethylene synthesis and signaling, indicate that GA biosynthesis is strongly regulated by GA and ethylene (Iglesias-Fernandez and Matilla, 2010).

Gibberellin signaling pathways depend on DELLA proteins including GAI (GA INSENSITIVE), RGA (REPRESSOR OF *ga1-3*), RGL1 (RGA LIKE1), RGL2 and RGL3 (Sun and Gubler, 2004; Davière and Achard, 2013). GAs destabilizes the DELLA proteins, which act as growth repressors by targeting GAs for ubiquitination and degradation (Dill et al., 2004). In *Arabidopsis*, Achard et al. (2003, 2007) reported that a part of ethylene action on hypocotyl growth and floral transition was mediated *via* its effects on the DELLA proteins. This may be true too in the control of germination since DELLA proteins seem to play a key role in the regulation of seed germination (Lee et al., 2002; Tyler et al., 2004; Cao et al., 2006; Steber, 2007; Piskurewicz et al., 2008; Schwechheimer, 2008). Thus, the seed GA content and responsiveness may result from a regulation of DELLA accumulation by ethylene.

## ROS AND ETHYLENE INTERACT TO REGULATE SEED GERMINATION

It has been shown in various seed species (Oracz et al., 2007; Ishibashi et al., 2008, 2013; Müller et al., 2009; Bahin et al., 2011), including *Arabidopsis* (Liu et al., 2010; Leymarie et al., 2012), that radicle protrusion is associated with and/or required a controlled accumulation of ROS. Regarding the role of plant hormones in seed germination and dormancy, several studies have investigated the possible relationship between metabolic and signaling pathways of these hormones, mainly ABA and GAs, and ROS homeostasis (Bailly et al., 2008). Up-to-date, however, the relationship between ROS and ethylene has been scarcely studied within the context of seed germination, although this is well documented in other contexts such as plant pathogen interactions (Mersmann et al., 2010) or cell death regulation (Overmyer et al., 2003).

In sunflower embryos, whose dormancy is released by exogenous ethylene (Corbineau et al., 1990), it has been recently demonstrated that ethylene markedly enhanced ROS accumulation within dormant embryonic axes, probably through the activation of NADPH oxidase (El-Maarouf-Bouteau et al., 2014). Whether ethylene produced in response to ROS has a direct effect on cell wall properties and cell elongation or if it stimulates cell signaling pathways related to germination, is however not known. Contrasting results obtained by Lin et al. (2012, 2013) demonstrates that ethylene decreases ROS content in *Arabidopsis* seeds germinating under salinity stress. In the case of the former species, ethylene has an antagonistic effect to ROS that are detrimental for germination, probably because their production increases to excessive levels in response to stress. This highlights the plasticity of seed responses to ROS but also the complexity of their interaction with ethylene (Bailly et al., 2008).

Several authors have also studied the effect of ROS on ethylene production during seed germination. Dormant sunflower embryos treated by methylviologen, a ROS generating compound, germinate rapidly at temperatures that would otherwise prevent their germination (Oracz et al., 2007). However, this improving effect is not associated with an increase in ethylene production which peaks at the time radicle elongates, and which therefore must be considered as a post-germinative event (El-Maarouf-Bouteau et al., 2014). These authors propose that ethylene might participate in association with ROS to facilitate the initiation of cell elongation, the first visible symptom of germination. In dormant apple embryos, Gniazdowska et al. (2010) suggest that the improving effect of NO and HCN results from a transient ROS production leading to an ethylene production required in termination of the *sensu stricto* germination process before radicle elongation and propose that this might result from a non-enzymatic oxidation of ACC. Ishibashi et al. (2013) have proposed that ROS produced in soybean (*Glycine max*) embryonic axes during imbibition induces ethylene production, which promotes cell elongation in the radicle. However, in that case, ethylene was measured after the onset of radicle protrusion, and this production was probably more related to the kinetics of seedling elongation than on a direct effect of H_2_O_2_. In contrast, in pea, which germination is not strongly regulated by ethylene, Barba-Espin et al. (2011) demonstrate that H_2_O_2_ treatment results in a reduction in *PsACS2* transcript abundance consistent with a decrease in ACC content. These results suggest that ROS and ethylene probably do not interact directly, but rather through a complex hormonal network (Diaz-Vivancos et al., 2013).

All together these data suggest that the interaction between ROS and ethylene in seeds can operate in both directions, depending on the physiological context of germination, i.e., on the environmental conditions prevailing during imbibition, and is highly species related. One can predict that the use of seeds of the plant model *Arabidopsis* will help decipher the molecular bases of this interaction. In particular it will be interesting to determine whether ROS can trigger expression of the ethylene signaling pathway components and *vice versa*. For example, Oracz et al. (2009) has demonstrated the occurrence of such a cross talk, since the treatment of dormant sunflower embryos by methylviologen induced the expression of ETR2 and of the transcription factor ERF1. The involvement of ethylene transcription factors in response to ROS appears to be worth investigating since studies with other plant systems have also implicated such a relationship (Sewelam et al., 2013).

## CONCLUSION: NETWORK BETWEEN ETHYLENE, PLANT HORMONES, AND ROS

**FIGURE 2:**
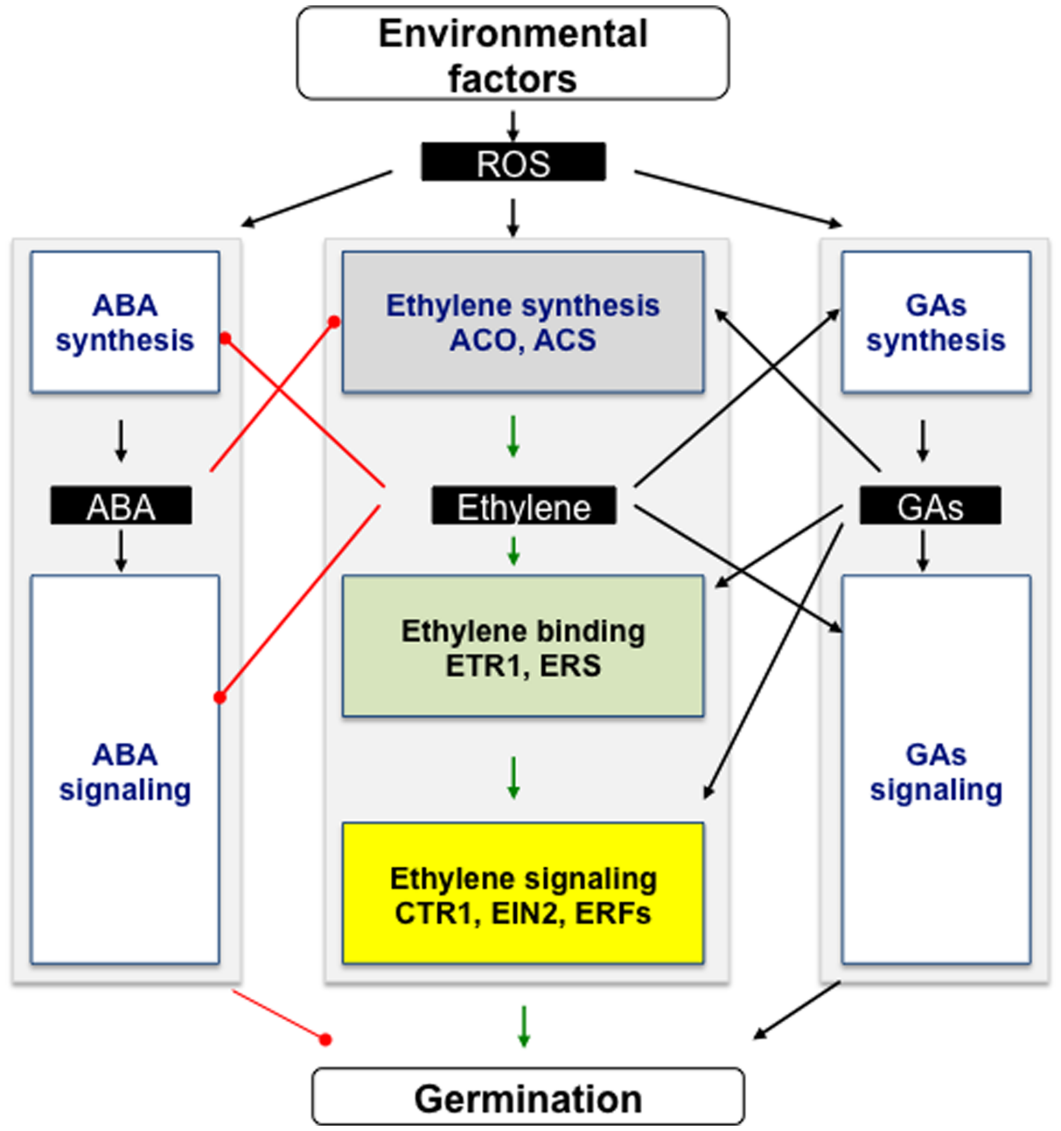
**Interactions between ethylene, abscisic acid, gibberellins, and ROS in the regulation of seed germination and dormancy.** This scheme is based on genetic analyses, microarray data, and physiological studies on seed responsiveness to ethylene, ABA, GAs, or ROS cited in the text. Ethylene down-regulates ABA accumulation by both inhibiting its synthesis and promoting its inactivation or catabolism, and also negatively regulates ABA signaling. ABA inhibits ethylene biosynthesis through ACS and ACO activities. Ethylene also improves the GAs metabolism, and GAs signaling, and *vice versa*. ROS enhance ABA catabolism and both C_2_H_4_ and GAs signaling. Whether ROS are signals induced by environmental factors to modulate the hormonal network toward germination is to be investigated. → and —∙ arrows indicate positive and negative interactions between the different elements of the signaling cascade, respectively.

Seed germination is regulated by ethylene in a complex signaling network, which is also operational in numerous developmental processes, including vegetative growth, flowering timing, fruit ripening and organ senescence and abscission (Yoo et al., 2009; Muday et al., 2012; Arc et al., 2013). As mentioned above, ethylene interacts with ABA and GAs, both hormones being essential regulators of germination and dormancy (Feurtado and Kermode, 2007; Nambara et al., 2010; Nonogaki et al., 2010; Miransari and Smith, 2014). Thus, the improving effect of ethylene may occur *via* the involvement of C_2_H_4_-GAs-ABA crosstalk but whether its action is direct or indirect needs clarification. Research on the effect of ABA and GAs on C_2_H_4_ biosynthesis and signaling pathways, especially in seeds, would then require further investigation, specifically in relation with ROS. **Figure 2** summarizes the current data concerning ABA-GAs-C_2_H_4_ networks based on genetic analyses, microarray data, and physiological studies. ABA inhibits the C_2_H_4_ biosynthesis pathway *via* an inhibitory action on ACO activity and on the *ACO* transcript accumulation. On the contrary, C_2_H_4_ counteracts both ABA synthesis and signaling, ETR1 having a key role. In addition, C_2_H_4_ affects the synthesis of GAs via modification of expression of genes (*GA3ox* and *GA20ox*) involved in GAs synthesis. Ethylene also probably modifies the GAs signaling pathway *via* a regulation of DELLA proteins, as demonstrated in growth processes (Achard et al., 2003, 2007). To add to the complexity of the ABA-GAs-C_2_H_4_ network, there are antagonistic interactions between ABA and GAs, C_2_H_4_ and brassinosteroids, jasmonates and auxins (Wang et al., 2002; Brady and McCourt, 2003; Weiss and Ori, 2007; Finkelstein et al., 2008; Matilla and Matilla-Vazquez, 2008; Cheng et al., 2009; Divi et al., 2010; Linkies and Leubner-Metzger, 2012). ROS also regulate seed germination through hormonal networks, in particular with ABA and GAs (Bethke et al., 2007; Liu et al., 2010; Diaz-Vivancos et al., 2013). It would be then important to discriminate the hierarchy of the different signaling pathways, and their role as sensor of environmental signals.

Omics studies are now available in the field of seed germination but efforts to develop transcriptomic analysis of ethylene action are required to understand ethylene involvement in seed germination. Analysis of the effects of ethylene on specific cellular processes highlighted by dormancy and germination studies such as transcription regulation, cell cycle activity and endosperm weakening should help to understand the regulatory network of germination process in seeds. Moreover, although hormonal signaling network share common components, they may work in specific territories in seeds.

## Conflict of Interest Statement

The authors declare that the research was conducted in the absence of any commercial or financial relationships that could be construed as a potential conflict of interest.
